# Azelaic Acid Exerts Antileukemic Activity in Acute Myeloid Leukemia

**DOI:** 10.3389/fphar.2017.00359

**Published:** 2017-06-12

**Authors:** Yunbao Pan, Dong Liu, Yongchang Wei, Dan Su, Chenyang Lu, Yanchao Hu, Fuling Zhou

**Affiliations:** ^1^Department of Laboratory Medicine, Zhongnan Hospital of Wuhan UniversityWuhan, China; ^2^Department of Clinical Pharmacy, Baoji Central HospitalBaoji, China; ^3^Department of Radiation and Medical Oncology, Zhongnan Hospital of Wuhan UniversityWuhan, China; ^4^Department of Clinical Hematology, The Second Affiliated Hospital, Xi’an Jiaotong UniversityXi’an, China; ^5^Department of Hematology, Zhongnan Hospital of Wuhan UniversityWuhan, China

**Keywords:** Azelaic acid, acute myeloid leukemia, Jab1, AKT, targeted therapy

## Abstract

Acute myeloid leukemia (AML) is an acute leukemia common in most adults; its prevalence intensifies with age. The overall survival of AML is very poor because of therapeutic resistance. Azelaic acid (AZA) is non-toxic, non-teratogenic, and non-mutagenic and its antitumor effect on various tumor cells is well established; Nonetheless, its therapeutic effects in AML cells are largely unknown. In this study, it was shown that AZA significantly inhibits the cell viability and induces apoptosis in AML cells in a dose-dependent manner. Additionally, AZA suppressed the expression of phosphorylated Akt, Jab1 and Trx, and this suppression was enhanced by treatment with Jab1 siRNA. Furthermore, AZA sensitized AML cells to Ara-c chemotherapy. The suppressive effect of AZA on tumor growth was examined *in vivo* by subcutaneously inoculated AML cells in a tumor model using nude mice. These findings indicate that AZA is useful as an effective ingredient in antineoplastic activity.

## Introduction

Acute myeloid leukemia (AML) is a cancer of the myeloid lineage of blood cells and it is the most common form of acute leukemia characterized by an unusual expansion of hematopoietic precursor cells with little or unusual differentiation that results in the accumulation of immature leukemic blasts. AML is responsible for almost 85% of adult patients and is a leading cause of cancer-related death (Deschler and Lubbert, 2006).

Significant progress in the development of novel targeted therapies and chemotherapeutic agents has improved the outcomes of AML patients (Yanada and Naoe, 2012). Nonetheless, the overall survival of AML patients remains low due to the therapy resistance and risk of developing therapy-related malignancy. It was reported that about 13.4% of AML patients that survived for more than 10 years developed secondary tumor (Micallef et al., 2001).

Cancer initiation mainly originates from uncontrolled malignant cell proliferation, deregulation of apoptosis. Cell death and survival are under modulation from a network of transmembrane and/or intracellular signals. One of such signal target is Jab1, which is participated in tumorigenesis by targeting many substrates, such as p27, cyclin E, Smad 4/7, p53, and so on (Pan et al., 2014). Indeed, Jab1 overexpression is inversely associated with p27 expression and with poor survival in different human cancers (Pan et al., 2014). In addition, overexpression of Jab1 is correlated with lymph node metastasis in cancer patients (Hsu et al., 2008; Gao et al., 2012). Our recent studies indicated Jab1 was overexpressed in AML and was associated with low overall survival rates in patients with AML. Mechanistically, Jab1 not only transcriptionally regulated Trx but also interacted with Trx and stabilized Trx protein. Moreover, depletion of Jab1 inhibited leukemia cell growth both *in vitro* and *in vivo* (Zhou et al., 2017). Therefore, Jab1 overexpression can be considered as a biomarker of poor prognosis in different human malignancies.

The phosphatidylinositol 3-kinase (PI3K)/Akt pathway mediates cell proliferation and other malignant properties in a variety of tumors. Inhibition of Akt always suppresses cell proliferation and induces cell apoptosis. The Akt phosphorylated by PI3K bolsters cell survival, proliferation, and probably other malignant phenotypes by phosphorylating downstream substrates such as transcriptional factors NF-κB and forkhead box protein (FOXO) (Brunet et al., 1999; Romashkova and Makarov, 1999; Vivanco and Sawyers, 2002). Significantly, increased phosphorylated Akt has been seen in a vast of malignancies (Mayer and Arteaga, 2016; Zhu et al., 2016). The link between Akt and cancer progression makes it a promising target for cancer therapy (Mayer and Arteaga, 2016). Development of drugs targeting Jab1 or Akt has become a promising strategy for cancer treatment.

Azelaic acid (AZA) is a naturally occurring, non-toxic, non-phenolic, non-teratogenic, saturated, non-mutagenic, straight-chained, saturated, and nine-carbon atom dicarboxylic acid isolated from cultures of *Pityrosporum ovale*, and possess antimicrobial (Gollnick and Schramm, 1998; Breathnach, 1999). AZA has been shown to exhibit antitumor effect on several cancer cells, such as human cutaneous malignant melanoma and human choroidal melanoma (Breathnach, 1999). However, the efficacy of AZA on AML cells is largely unknown. Thus, we investigated the antileukemic activity of AZA in AML cells.

## Materials and Methods

### Materials

Cell culture medium and fetal bovine serum were purchased from Invitrogen (Carlsbad, CA, United States). Antibodies to the following proteins were used: PARP (BD Biosciences, San Jose, CA, United States, Cat#556494); Jab1 (Santa Cruz, CA, United States, Cat#sc-13157); p-Akt (p-Thr308) (Cell Signaling, Danvers, MA, United States, Cat#13038), T-Akt (Cell Signaling, Danvers, MA, United States, Cat#2920) and β-actin (Cell Signaling, Danvers, MA, United States, Cat#3700). Western Lightning Chemiluminescence Plus reagent was from Thermo Scientific Pierce (Rockford, IL, United States, Cat# 34087). Annexin V/PI kit was from BD (Cat# 556547). AZA (Cat# 95054), DMSO (Cat# D2650), and 3(4,5)dimethylthiahiazo(zy1)3,5diphenytetrazoliumromide (MTT) (Cat# M2128) were from Sigma.

### Cell Culture

Human AML U937, THP-1, KG-1, NB4, and HL-60 cells were purchased from ATCC (Manassas, VA, United States), and were grown in RPMI-1640 medium supplemented with 10% fetal bovine serum and 2 mmol/L L-glutamine. Cell lines were maintained between 2 × 10^5^ and 1 × 10^6^ cells/mL, and cell viability was analyzed before each assay using a trypan blue dye exclusion assay (using viability >96%). Peripheral blood mononuclear cells (PBMC) were isolated from peripheral blood of healthy donors by differential density gradient centrifugation of blood collection. Briefly, blood was diluted 1:1 in phosphate-buffered saline (PBS) at RT prior to layering over Lymphocyte Separation Medium (TBD science, Tianjin, China, Cat# HY2015). PBMC were collected following centrifugation (800*g*, 20 min) and washed in PBS (320*g*, 10 min). Cells were resuspended in RPMI-1640 medium supplemented with 10% fetal bovine serum. This study was carried out in accordance with the recommendations of Ethics and Scientific Committee of Zhongnan Hospital of Wuhan University with written informed consent from all subjects. All subjects gave written informed consent in accordance with the Declaration of Helsinki.

### Determination of Cell Viability by MTT Assay

MTT assay was performed as described previously (Pan et al., 2013). AML cells were seeded in 96 well plates and exposed to 0.1–50 mmol/L AZA for 24–72 h. Cells were labeled with 20 μL MTT labeling reagent, and the absorbance was determined on a spectrophotometric microplate reader (Bio-Rad, Hercules, CA, United States). The optical densities were measured at 570 nm. Results were calculated as percentage of unexposed control.

### Colony Formation Assays

Clonogenic assays were used to investigate cell proliferation (Zhou et al., 2017). Briefly, 2 mL medium with 0.7% agar was added in each well of a six-well plate. After the bottom agar solidified, another 1 mL 0.35% agar in medium containing 5,000 cells was added. The cells were exposed to AZA at indicated concentration for 24 h. Fourteen days later, the finally colonies consisting of more than 50 cells were counted.

### Determination of Cell Viability by Alamar Blue Assay

Cell viability was examined using the alamar blue assay. AML cells were seeded in 96-well cell culture plates at a density of 1 × 10^4^ cells/well. AML cells were treated with Ara-c (5 μM), AZA, or combined with AZA. After being treated for 24 h, 10 μL of Alamar Blue was then added to each well and then the plates were incubated for measurement at different time points. Thereafter, all plates were measured at 560 nm.

### Small Interfering RNA (siRNA) Transfection

Cells were transfected using Nucleofector Kit V from Amaxa Biosystems (Lonza, Allendale, NJ, United States). The control siRNA (Cat#sc-37007) and human Jab1-siRNA (Cat#sc-35717) were obtained from Santa Cruz Biotechnology (Dallas, TX, United States) and were transfected by electroporation (Zhou et al., 2017).

### Measurement of Apoptosis

Apoptotic cells were examined by Annexin V-FITC and PI staining as described previously (Pan et al., 2013). Briefly, after AZA treatment for 24 h, cells were labeled with Annexin V and PI according to manual. Quantification of apoptosis cells was analyzed by flow cytometry (BD Biosciences).

### Cell Extracts and Immunoblotting

Protein was extracted by RIPA buffer and were subjected to sodium dodecyl sulfate-polyacrylamide gel electrophoresis and transferred to a polyvinylidene difluoride membrane (Zhou et al., 2017). Antibodies against the following proteins were used: PARP, Jab1, p-Akt, T-Akt. β-actin served as a control for protein load and integrity in all immunoblots.

### Tumorigenicity Assay in Nude Mice

Animal work was conducted as previously described (Zhou et al., 2017). Four-week-old athymic nude (nu/nu) mice obtained from the Animal Research Center of Wuhan University received subcutaneous injections of 2 × 10^6^ U937 cells in the right flank. Mice were checked every 2 days for xenograft development. Treatment—intraperitoneal injections of 0.5 g/kg AZA once every 2 days—was started after the tumors became palpable (about 0.1 mm^3^). Tumor volumes and mice’s body weights were measured twice weekly. Tumor volume (in cubic millimeters) was calculated as tumor length × tumor width^2^/2. At the end of the experiments, the mice were humanely killed under general anesthesia; the tumors were excised and weighed. All animal experiments were carried out under protocols approved by the Institutional Animal Care and Use Committee of Wuhan University (2017048).

### Statistical Analysis

Data were expressed as means ± SD; Student’s *t*-test and analysis of variance (ANOVA) were carried out among groups, and the level of statistical significance was set at *P* < 0.05. The calculations were performed using IBM SPSS Statistics software, 16.0.

## Results

### AZA Inhibits Cell Viability in AML Cells

**FIGURE 1:**
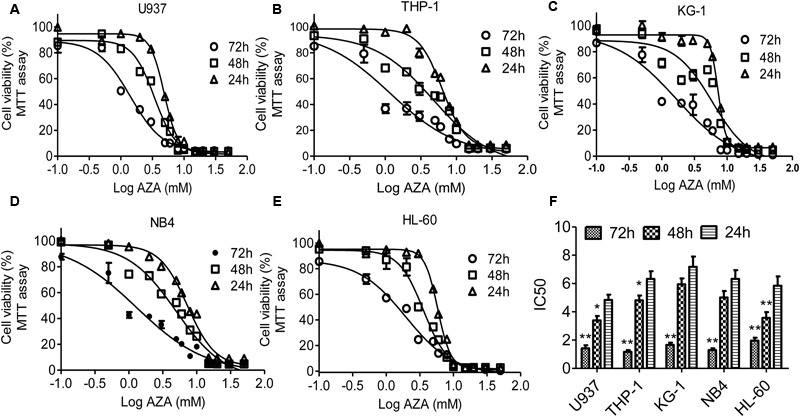
AZA inhibited viability of AML cells. AML cells U937 **(A)**, THP-1 **(B)**, KG-1 **(C)**, NB4 **(D)** and HL-60 **(E)** were incubated with AZA and cell viability was then measured by the MTT assay. **(F)** The concentration of drug required to obtain 50% maximal inhibition in cell viability was indicated by IC_50_. The data are the means with standard deviations for three independent experiments. Compared with 24 h (ANOVA), ^∗^*P* < 0.05, ^∗∗^*P* < 0.01.

In this study, the antileukemic activity of AZA in AML cells was examined using MTT assay. AZA suppresses the cell viability of five AML cells with time- and does-dependent manner (**Figure 1**). IC_50_ values of AZA were 1.4 mM for 72 h, 3.4 mM for 48 h and 4.8 mM for 24 h in U937, which were substantially the same as that in THP-1 cells (IC_50_ values 1.2, 4.8, and 6.3 mM, respectively) and KG-1 cells (IC_50_ values 1.7, 5.9, and 7.2 mM, respectively) and NB4 cells (IC_50_ values 1.3, 5.1, and 6.3 mM, respectively) and HL-60 cells (IC_50_ values 1.9, 3.6, and 5.8, respectively). The IC_50_ value of 72 h is significant lower compared with those of 24 h (**Figure 1D**).

### AZA Inhibits Cell Growth and Induces Apoptosis in AML Cells

**FIGURE 2:**
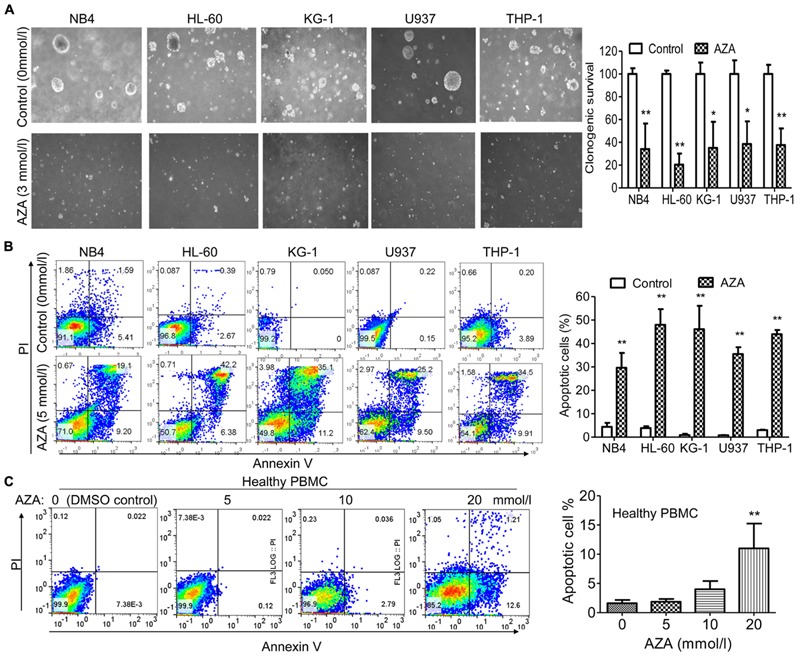
AZA inhibited cell proliferation and induced apoptosis in AML cells. **(A)** Left, representative results of colony formation assays with AML cells treated with AZA. Right, quantification of colonies. AML cells **(B)** or PBMC **(C)** from healthy donors were treated with DMSO control or AZA for 24 h and then stained with Annexin-V/PI and examined by flow cytometry. Right, quantification of apoptotic cells. The data are means with standard deviations for three independent experiments. Compared with “control” group (student’s *t*-test) or “0” group (ANOVA), ^∗^*P* < 0.05, ^∗∗^*P* < 0.01.

Treatment of AML cells with AZA led to decreased colony formation when compared to the DMSO control group (**Figure 2A**). Three mmol/L of AZA produced a decrease of nearly 66% (NB4), 80% (HL-60), 65% (KG-1), 61% (U937) and 62% (THP-1) in colony formation, respectively. These results indicated that AZA was potent in inhibiting cell growth of AML cell lines.

We next examined the effect of AZA on the induction of apoptosis using Annexin V and PI dual staining. After 5 mmol/L of AZA treatment for 24 h, the apoptosis rates were 30% (NB4), 48% (HL-60), 46% (KG-1), 36% (U937) and 44% (THP-1), respectively (**Figure 2B**). Interestingly, we did not observe apoptosis in healthy PBMC cells after AZA treatment even at the concentration of 10 mmol/L (**Figure 2C**), suggesting AZA is specific to AML cells.

### AZA Enhances the Antileukemic Effects of Ara-c in AML

**FIGURE 3:**
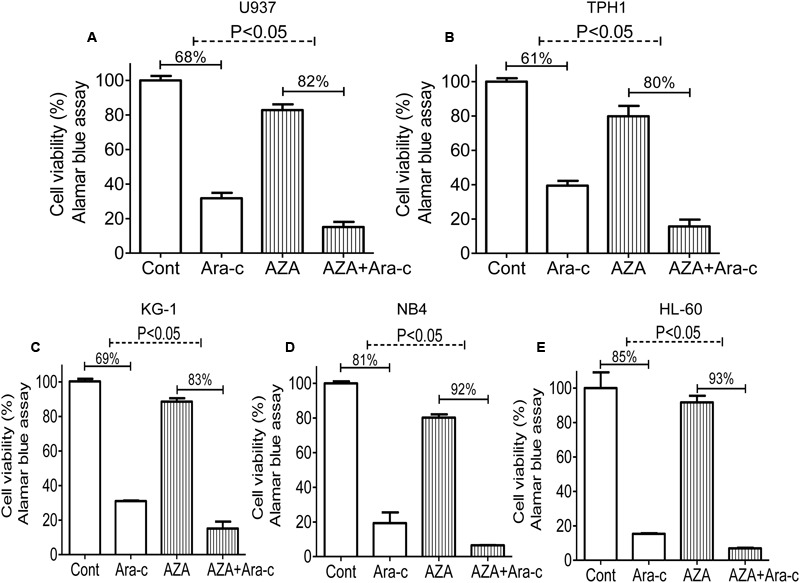
AZA sensitized AML cells to Ara-c chemotherapy. AML cells [U937 **(A)**, TPH1 **(B)**, KG-1 **(C)**, NB4 **(D)**, HL-60 **(E)**] were treated with Ara-c (5 μmol/L) alone or together with AZA (1 mmol/L) for 24 h and the cell viability were detected using Alamar Blue assay. The data are means with standard deviations for three independent experiments, statistical significance was determined with student’s *t*-test. Cont, control.

Ara-c is a clinical drug for AML, we investigated if AZA is participated in the antileukemic activity of Ara-c. Sub-optimal dose (<IC_20_) of AZA was used to investigate whether AZA sensitize AML cells to Ara-c (5 μmol/L) using the alamar blue assay. As predicted, AML cells with AZA treatment had a significant higher Ara-c efficacy than control cells treated with Ara-c alone. The Ara-c exerted antitumor effect and showed cell viability inhibition in all the five AML cells tested (68% in U937, 61% in THP1, 69% in KG-1, 80% in NB4, and 84% in HL-60, respectively), while cell viability was more reduced following combined treatment with AZA and Ara-c (85% in U937, 84% in THP1, 85% in KG-1, 94% in NB4, and 93% in HL-60, respectively) (**Figure 3**).

### AZA Inhibits the Expression of Jab1 and Akt in AML Cells

**FIGURE 4:**
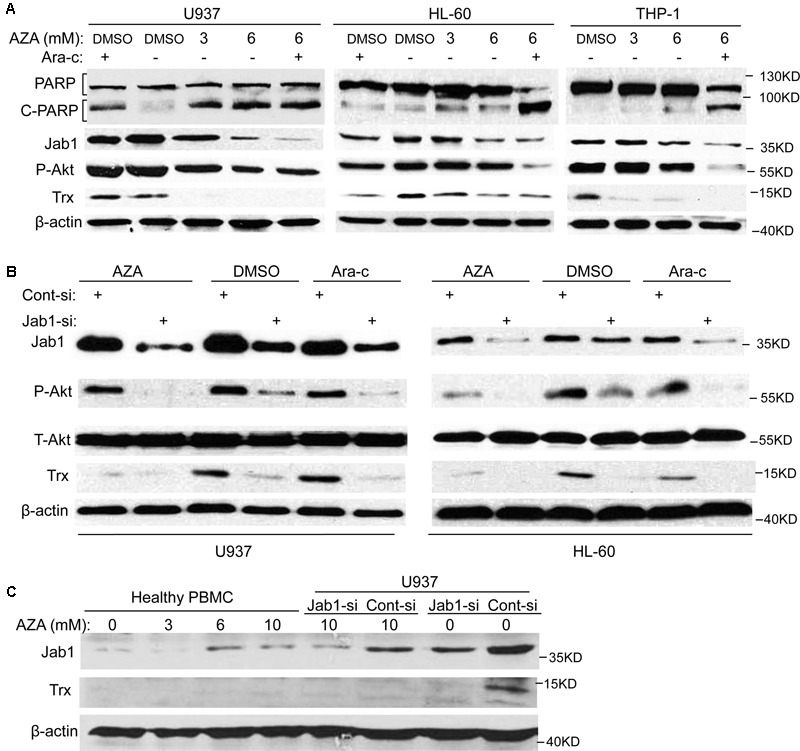
AZA inhibited Jab1 in AML cells. **(A)** AML cells were treated with different dose of AZA or combined with Ara-c (2.5 μmol/L) for 24 h, and then lysed for western blotting. **(B)** AML cells were transfected with Jab1 siRNA for 48 h, and then exposed to AZA (3 mmol/L) or Ara-c (2.5 μmol/L) for 24 h, specific protein expression were detected by western blotting. **(C)** PBMC from healthy donors or U937 cells with knockdown of Jab1 were treated with AZA at the indicated concentration for 24 h and then lysated for western blotting.

Since cleavage of poly (ADP-ribose) polymerase (PARP) are indications of apoptosis (Lazebnik et al., 1994), the effect of AZA on PARP was further examined. As predicted, cleavage of PARP from 116 to 85 kDa was clearly elucidated after AZA and Ara-c treatment in all the AML cells tested (**Figure 4A**). In order to further elicit the signaling mediating the pro-apoptotic effect of AZA on AML cells, Jab1 and its target Trx, and Akt expression were also explored. It was found that AZA inhibited Jab1/Trx expression in a dose-dependent manner, while combined treatment of AZA and Ara-c resulted in most decrease in Jab1/Trx and phosphorylated Akt. The effect of AZA and Ara-c on these three proteins by pre-treating the AML cells with Jab1 siRNA was further explored. It was found that the decrease of phosphorylated Akt was induced by the reduction of Jab1, and this decrease was enhanced by additional treatment with either AZA or Ara-c (**Figure 4B**). In contrast, AZA slightly stimulated Jab1 expression in healthy PBMC (**Figure 4C**). These data indicated that the antileukemic effect of AZA in AML cells was mediated through Jab1 and associated with Akt status.

### Jab1 Associates with AZA Efficacy

**FIGURE 5:**
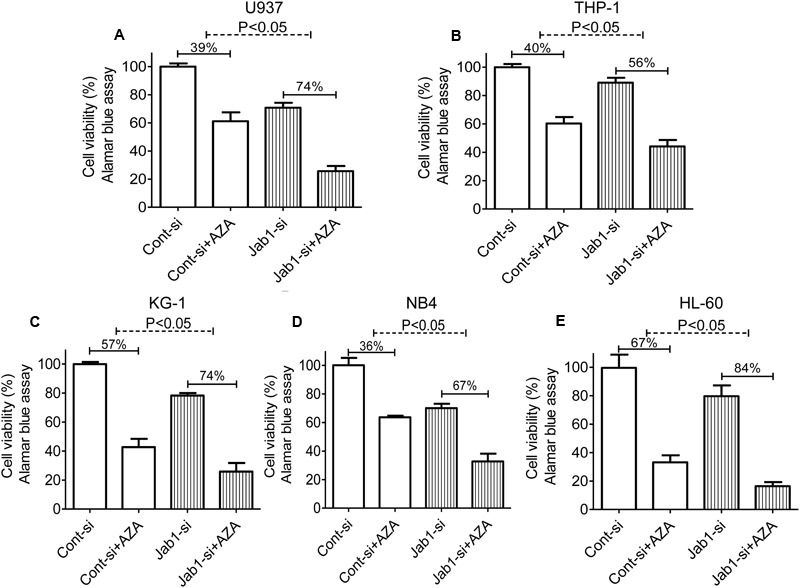
Depletion of Jab1 sensitized AML cells to AZA. AML cells [U937 **(A)**, THP-1 **(B)**, KG-1 **(C)**, NB4 **(D)**, HL-60 **(E)**] were treated with Jab1 siRNA for 48 h and were incubated with AZA (5 mmol/L) for 24 h, the cell viability was then determined by Alamar Blue assay. Cont-si, Control siRNA; Jab1-si, Jab1 siRNA. The data are means with standard deviations for three independent experiments, statistical significance was determined with student’s *t*-test.

In order to illuminate the role of Jab1 in AZA’s antileukemic activity, it is important to determine if suppression of Jab1 increase AZA efficacy. Cell viability of AML cells with Jab1 knockdown mediated by Jab1 siRNA was measured by Alamar Blue assay. AZA induced a significant higher cell viability inhibition in Jab1 deficient cells (74% in U937, 56% in TPH1, 74% in KG-1, 67% in NB4, and 84% in HL-60) compared with that observed in control cells (39% in U937, 40% in TPH1, 57% in KG-1, 36% in NB4, and 67% in HL-60) (**Figure 5**).

### AZA Represses Tumorigenicity of AML Cells

**FIGURE 6:**
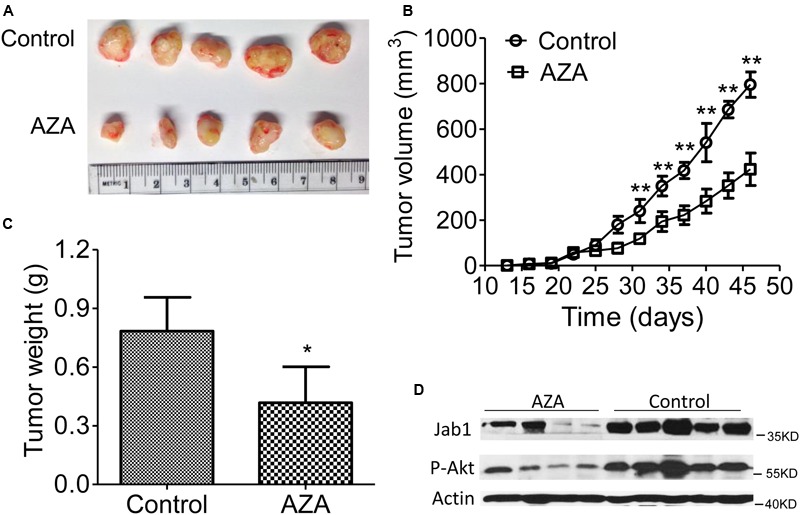
AZA inhibits AML tumor xenografts in athymic nude mice. **(A–C)** Representative photographs of harvested tumors **(A)** and the corresponding tumor growth curves **(B)** are shown. Tumor weights measured at the indicated times are shown **(C)**. The expression levels of Jab1 and P-Akt proteins in tissues resected from the same mice were examined via Western blot analysis **(D)**. Female nude mice bearing xenograft tumors derived from U937 were intraperitoneally injected with phosphate-buffered saline or AZA once every 3 days. At the end of the experiments, the mice were humanely killed, and the tumors were excised and weighed. The data are means with standard deviation. Compared with “control” group (student’s *t*-test), ^∗^*P* < 0.05, ^∗∗^*P* < 0.01.

We then transplanted the U937 cells into nude mice and treated the mice with AZA once tumors became palpable (**Figures 6A–C**). The suppressive effects of AZA on tumor growth were evident in the U937 mouse model. The injection of AZA substantially reduced tumor growth. Tumor weight was consistently significantly lower in the AZA-treated groups than in the control group (**Figure 6C**). A Western blot analysis showed that Jab1 and p-Akt expression were abundantly expressed in the control tumors, whereas the levels of both were reduced in the AZA treated tumors (**Figure 6D**). These data suggest that AZA suppresses AML *in vivo*.

## Discussion

Although progress in therapeutic approaches for AML patients, the overall survival of AML remains poor due to therapy resistance (Schoch et al., 2001). Therefore, identification of therapeutic drugs with less cytotoxicity is of great interest.

It has been demonstrated that AZA have anti-proliferative and cytotoxic action on many cancers (Schulte et al., 2015), such as human cutaneous malignant melanoma (Zaffaroni et al., 1990), human choroidal melanoma (Breathnach et al., 1989), human squamous cell carcinoma (Patzold et al., 1989), and leukemia derived cell lines (Picardo et al., 1985). AZA acts specifically against tumor cells; however, normal cells are unaffected at the same dosage (Breathnach, 1999). These properties in combination with its low toxicity have allowed itself as a promising antitumor candidate. Consistent with these studies, the antileukemic activity of AZA in different types of AML cells was investigated and it was found that AZA suppressed the cell viability of all the tested AML cell lines. The cell viability-suppressive potential of AZA in AML cells was associated with activation of apoptosis. NB4 cell line is positive for translocation *t*(15;17) (Lanotte et al., 1991) while HL-60 cell line is negative for translocation *t*(15;17) (Collins et al., 1977). Antileukemic activity was seen in both cell lines, suggesting that this translocation is not required for AZA efficacy.

Akt controls cell survival against the death program, and hyperactivation of the Akt pathway contributes to chemotherapy resistance in malignancies (Wang et al., 2016; Zhang et al., 2016). Activation of Thr308 on Akt also correlates with poor prognosis in AML patients (Gallay et al., 2009). However, development of safe therapeutic inhibitors of Akt is still challenging. Our studies have showed that AZA inactivated Akt on the Thr308 residue, and initiated the apoptotic poly PARP activation in AML cells. Although in-depth studies are required to illuminate whether the AZA directly or indirectly inhibits Akt, this study suggested that AZA was a promising anti-Akt and anti-cancer candidate. In addition, studies have suggested that AZA acts as a TRX (thioredoxin) reductase inhibitors and has a potential therapeutic usefulness for treatment of HTLV-1(+) T-cell leukemia (U-Taniguchi et al., 1995). Consistently, the decrease in TRX induced by AZA in AML cells was observed.

Our previous studies showed Jab1 is overexpressed in different types of cancer and it contributes to chemotherapy and radiotherapy resistance (Pan et al., 2013). In this study, the first evidence of AZA activity against AML by suppressing Jab1 was presented. It was observed that AZA was powerful in suppressing Jab1 signaling. With these findings, the potential influence of Jab1 on the activity of AZA was examined. Suppressing Jab1 enhanced the ability of AZA against AML cells. Furthermore, the data indicated that the AZA mediated decrease in p-Akt and Trx was enhanced by Jab1 siRNA treatment, suggesting a relationship between Jab1, Akt and Trx. This relationship is under study.

In addition, the cytotoxic effect of AZA alone or combined with Ara-c on AML cells have been addressed. AZA had a dose-dependent cytotoxic effect on all cell lines. By modulating multiple signaling pathways, AZA may sensitize AML cells to chemotherapy. The finding from this study suggests that a combination of AZA with Ara-c is more effective in treating AML than using either drug alone. The drug combination seems to overcome resistance to the single agents in AML cells.

## Conclusion

Our study indicated that AZA inhibited the expression of Jab1 and p-Akt in AML cells and enhanced the antileukemic activity of Ara-c. Thus, our data suggested that combination of AZA and Ara-c may potentially be used in development of promising agents.

## Author Contributions

YP and FZ conceived the study. YP, DL, YW, DS, CL, and YH performed the experiments. FZ directed research. YP and FZ analyzed the data and wrote the manuscript.

## Conflict of Interest Statement

The authors declare that the research was conducted in the absence of any commercial or financial relationships that could be construed as a potential conflict of interest. The reviewer VP and handling Editor declared their shared affiliation, and the handling Editor states that the process nevertheless met the standards of a fair and objective review.
